# First-Time Users of ADHD Medication Among Children and Adolescents in Germany: An Evaluation of Adherence to Prescribing Guidelines Based on Claims Data

**DOI:** 10.3389/fpsyt.2021.653093

**Published:** 2021-04-15

**Authors:** Oliver Scholle, Bianca Kollhorst, Oliver Riedel, Christian J. Bachmann

**Affiliations:** ^1^Department of Clinical Epidemiology, Leibniz Institute for Prevention Research and Epidemiology – BIPS, Bremen, Germany; ^2^Department of Biometry and Data Management, Leibniz Institute for Prevention Research and Epidemiology – BIPS, Bremen, Germany; ^3^Department of Child & Adolescent Psychiatry, University Hospital Ulm, Ulm, Germany

**Keywords:** ADHD, adolescents, children, pharmacotherapy, off-label use, pharmacoepidemiology

## Abstract

**Background:** Drug utilization studies based on real-world data are vital for the identification of potentially needed improvements to rational prescribing. This is particularly important for the pharmacological treatment of children and adolescents with attention-deficit hyperactivity disorder (ADHD) due to the associated potential side effects and the frequent use. Whereas prevalent use is well-characterized, studies on first-time use of ADHD medication are scarce. This study aimed to evaluate off-label prescribing in first-time users of ADHD medication among children and adolescents in Germany based on three criteria: (i) lack of a documented ADHD diagnosis; (ii) first-time pharmacological treatment with a second-line drug; and (iii) patient age below 6 years.

**Methods:** Based on German claims data, we included children and adolescents aged 0–17 years with a first-time dispensation of any ADHD medication in the period 2015–2017. These first-time users were characterized with regard to sex, age, specialty of the prescribing physician, documentation of an ADHD diagnosis, psychiatric hospitalization, psychiatric comorbidities, and history of other psychopharmacological drugs at first-time use.

**Results:** The study population comprised 18,703 pediatric first-time users of ADHD medication. Of these, 9.8% had no documented ADHD diagnosis. Most of the ADHD drug users received first-line ADHD pharmacotherapy (methylphenidate, atomoxetine), whereas 2.6% were prescribed second-line ADHD medication (lisdexamfetamine, guanfacine, dexamfetamine, multiple ADHD drugs) as first drug. Overall, 1.2% of first-time users were aged below 6 years. A total of 12.7% of the study population met any off-label criterion.

**Conclusions:** About 13% of pediatric first-time users of ADHD medication in Germany received an off-label pharmacotherapy at first-time use. Prescribing ADHD medication without a confirmed ADHD diagnosis was the most common of the three assessed off-label criteria. Off-label prescribing regarding drug choice and age of patients only occurred in a small percentage of initial pharmacological ADHD treatment. Our results suggest the need for improvement in rational prescribing, especially with regard to diagnostic requirements.

## Introduction

With a worldwide community prevalence between 2 and 7%, attention-deficit hyperactivity disorder (ADHD) is one of the most common mental disorders among children and adolescents (1, 2). The global burden of ADHD is significant (3, 4) and it is estimated that more than 40% of individuals with childhood ADHD continue to experience symptoms and impairment in adulthood (5). National and international guidelines on ADHD recommend a multimodal treatment approach for children and adolescents with a combination of medication and psychosocial interventions (6). Regarding short-term efficacy, current evidence supports pharmacological treatment, particularly stimulants, as the most efficacious ADHD treatment (7). The evidence for long-term effects of drugs to treat ADHD on reducing impairments such as educational outcomes is limited and inconsistent (8). There is a strong evidence base that treatment with ADHD medications reduces negative outcomes such as injuries, cigarette smoking, suicide, and criminal activity (4).

Before initiating medication, the prescriber must ensure that the patient has a confirmed diagnosis of ADHD. Current clinical guidelines recommend a full clinical interview including structured and comprehensive assessments for the ADHD diagnosis (6, 9). In addition to this, stimulants such as methylphenidate (MPH) and lisdexamfetamine (LDX) are basically exempt from reimbursement by statutory health insurance providers in Germany unless strict and comprehensive diagnostic requirements have been fulfilled (10).

Although the evidence base is the same, the approval status and guideline recommendations differ between countries in North America and Europe, particularly regarding LDX. In Germany—as in other European countries—only MPH and atomoxetine (ATX) are approved as the initial—i.e., first-time—pharmacological ADHD treatment without restriction and—in contrast to the approval in, e.g., the US and Canada—LDX (available since June 2013) and dexamfetamine (DEX) require insufficient response to previous MPH treatment. Similarly, guanfacine (GUA; available since January 2016) may be indicated only if stimulants such as MPH are not suitable. In its recommendations, the German guideline on ADHD points out that the approval status of the medication should be taken into account (11).

All mentioned drugs are not approved for children aged below 6 years, i.e., preschool children in Germany. Guidelines do not preclude pharmacological treatment for preschool children but emphasize that psychosocial interventions should be considered first. Medication should only be prescribed to children with residual symptoms and after an individual risk-benefit assessment.

Little is known about adherence to guidelines for ADHD medication prescribed to children and adolescents in routine care. Especially recent drug utilization studies from Europe and including all available ADHD medication are lacking. These are, however, important since LDX and GUA have only been available for a relatively short time in European countries. Early monitoring and identification of characteristics associated with off-label prescribing of these newer drugs is crucial as the safety of newer drugs in routine care is generally not well-understood.

Therefore, this study aimed to evaluate off-label prescribing in first-time users of ADHD medication among children and adolescents in Germany based on three criteria: (i) lack of a documented ADHD diagnosis; (ii) first-time pharmacological treatment with a second-line drug; and (iii) patient age below 6 years.

## Materials and Methods

### Data Source

This study used data from the German Pharmacoepidemiological Research Database (GePaRD) (12). GePaRD is a claims database which includes information on persons who have been insured with one of the four participating statutory health insurance providers since 2004 or later. Per data year, GePaRD covers information on ~20% of the general population of Germany. About 90% of the general population are covered by statutory health insurance in Germany and there is a free choice of providers (13). Children are typically covered with one parent or legal guardian without any surcharges.

In addition to demographic data, GePaRD contains information on reimbursable drug dispensations as well as outpatient (i.e., from general practitioners and specialists) and inpatient services and diagnoses. Drug dispensations are identifiable via the German modification of WHO Anatomical Therapeutic Chemical (ATC) classification codes. Diagnoses are coded according to the German Modification of the 10th revision of the International Statistical Classification of Diseases and Related Health Problems (ICD).

### Study Population

We included children and adolescents aged 0–17 years with a dispensation of any ADHD medication between January 1, 2015 and December 31, 2017 and with health insurance coverage on the earliest dispensation date in that period. ADHD medication included all drugs approved to treat ADHD in Germany at the time, which were identified based on ATC codes: MPH (N06BA04), ATX (N06BA09), LDX (N06BA12), DEX (N06BA02), or GUA (N06BA21; only since January 2016).

Individuals were excluded if they did not have a minimum pre-observation time (i.e., health insurance coverage) of 4 years before the earliest dispensation date; those aged 4 years or younger were required to have a pre-observation time since the year of birth. Next, we excluded all individuals with a dispensation date of any ADHD medication in the pre-observation period (i.e., prevalent users).

The final study population can therefore be considered as first-time users of any ADHD medication. Depending on the ADHD medication(s) dispensed on the day of first-time use, each individual was assigned to one of six mutually exclusive groups of users: MPH; ATX; LDX; DEX; GUA; or users of more than one of these drugs.

### Characteristics of the Study Population

Characteristics assessed for each individual of the study population included year of first-time use, sex, and age. We examined whether ADHD (ICD-10 codes F90/F98.8) and/or narcolepsy (for which some MPH preparations are licensed in Germany; ICD-10 code G47.4) had been coded in the 2 years before and including the day (inpatient data) or quarter (outpatient data) of first use. The specialty of the prescribing physician was derived from the prescription. Psychiatric hospitalizations were identified based on hospital admissions with at least one ICD-10 code F00–F99 as main or secondary discharge diagnosis in the year before and including the day of first use. Psychiatric comorbidities were assessed from inpatient data in the year before and including the day of first use; in outpatient data—as outpatient diagnoses are recorded quarterly—psychiatric comorbidities were assessed in the quarter of the day of first use and in the three preceding quarters. History of other psychopharmacological drugs—antipsychotics (ATC codes starting with N05C), anxiolytics (N05B), hypnotics and sedatives (N05C), and antidepressants (N06A)—was assessed in the year before (not including) the day of first use of ADHD medication.

### Data Analysis

Descriptive analyses were conducted in first-time users overall and stratified by (i) whether or not there was a lack of a documented ADHD diagnosis, (ii) whether or not second-line ADHD medication was dispensed on the day of first use, and (iii) whether or not the age was below 6 years.

We additionally evaluated characteristics associated with (off-label) prescribing of a second-line pharmacological treatment as the first ADHD medication. Multivariable logistic regression was used to obtain odds ratios (OR) and corresponding 95% confidence intervals (CI) for the association between the characteristics described above and the prescribed line of treatment, comparing first-time users of second-line drugs (LDX, DEX, or GUA) with those of first-line drugs (MPH or ATX). We did not include all psychiatric comorbidities as independent variables but rather selected those deemed clinically relevant for the decision-making process regarding the prescription of ADHD medication.

## Results

**Figure 1:**
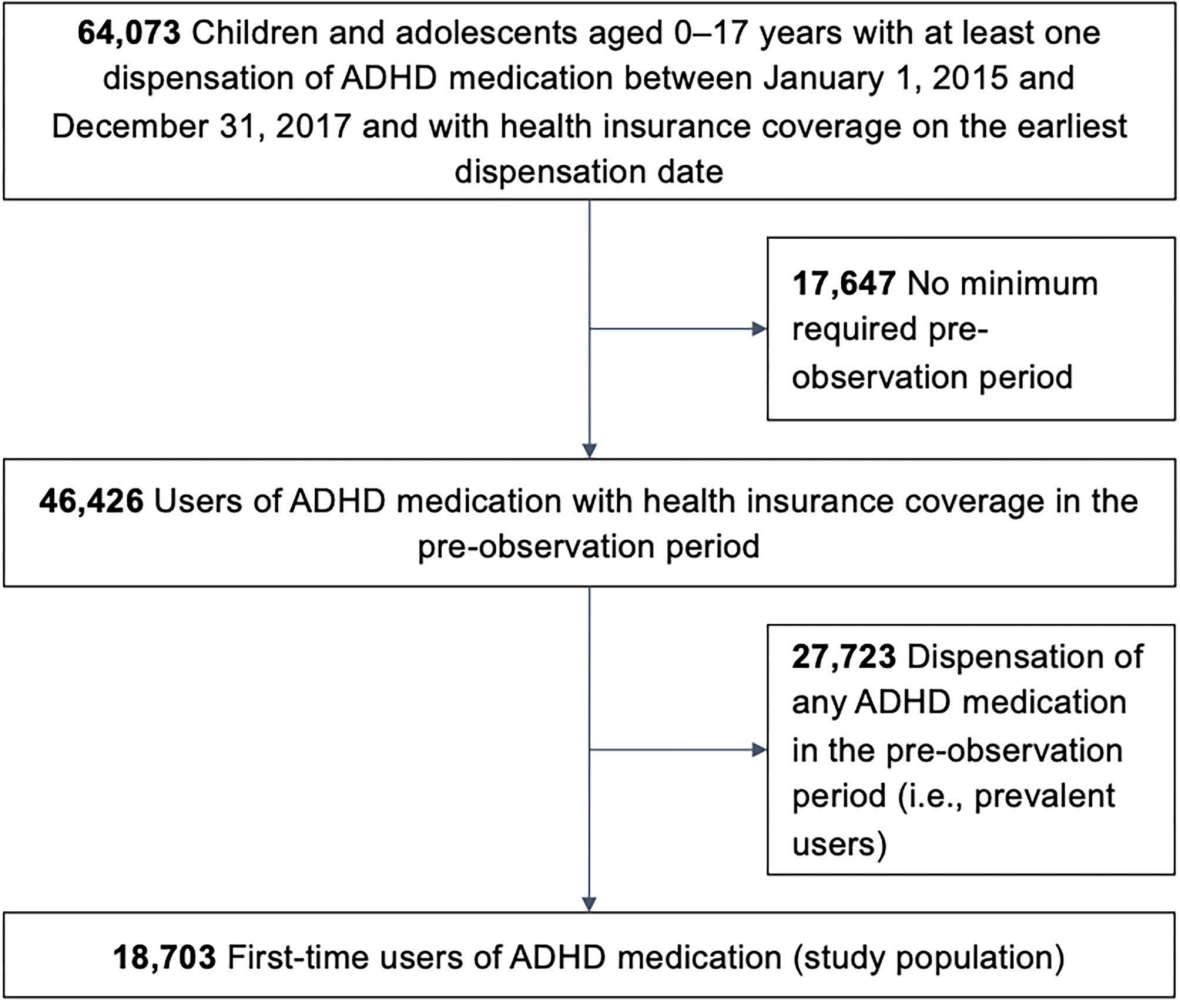
Flow diagram. ADHD, attention deficit hyperactivity disorder.

The study population comprised 18,703 pediatric first-time users of ADHD medication (Figure 1). Overall, 75% of all first-time users were male (Table 1). Any one of the three off-label criteria was fulfilled by 12.7% of the study population. For 9.8%, there was no documented diagnosis of ADHD. This patient group encompassed 0.1% of patients with a diagnosis of narcolepsy without ADHD and 9.7% without either a diagnosis of ADHD or narcolepsy. The most commonly prescribed ADHD medication was MPH, followed by ATX; multiple drugs were dispensed to 19 individuals (0.1%). A total of 2.6% of all individuals were prescribed second-line ADHD drugs as first pharmacological treatment. Overall, 1.2% of ADHD medication users were younger than 6 years.

**Table 1:** Characteristics of first-time users of ADHD medication, overall and by off-label prescribing criteria.

		**Lack of a documented ADHD diagnosis**		**Second-line drug as first ADHD medication^+^**		**Patient age below 6 years**	
**Characteristic**	**Overall (*n* = 18,703)**	**No (*n* = 16,874)**	**Yes (*n* = 1,829)**	**No (*n* = 18,218)**	**Yes** **(*n* = 485)**	**No (*n* = 18,483)**	**Yes** **(*n* = 220)**
**Sex**							
Female	4,634 (24.8)	4,039 (23.9)	595 (32.5)	4,501 (24.7)	133 (27.4)	4,590 (24.8)	44 (20.0)
Male	14,069 (75.2)	12,835 (76.1)	1,234 (67.5)	13,717 (75.3)	352 (72.6)	13,893 (75.2)	176 (80.0)
**Documented diagnosis**							
Ever ADHD (F90, F98.8)	16,874 (90.2)			16,486 (90.5)	388 (80.0)	16,700 (90.4)	174 (79.1)
Narcolepsy (G47.4) without ADHD	21 (0.1)			20 (0.1)	1 (0.2)	20 (0.1)	1 (0.5)
None of the above	1,808 (9.7)			1,712 (9.4)	96 (19.8)	1,763 (9.5)	45 (20.5)
**Age group in years**							
<6	220 (1.2)	174 (1.0)	46 (2.5)	200 (1.1)	20 (4.1)		
6–11	12,661 (67.7)	11,599 (68.7)	1,062 (58.1)	12,402 (68.1)	259 (53.4)		
12–17	5,822 (31.1)	5,101 (30.2)	721 (39.4)	5,616 (30.8)	206 (42.5)		
**ADHD medication**							
MPH	17,656 (94.4)	15,999 (94.8)	1,657 (90.6)			17,465 (94.5)	191 (86.8)
ATX	562 (3.0)	487 (2.9)	75 (4.1)			553 (3.0)	9 (4.1)
LDX	261 (1.4)	222 (1.3)	39 (2.1)			254 (1.4)	7 (3.2)
DEX	41 (0.2)	36 (0.2)	5 (0.3)			34 (0.2)	7 (3.2)
GUA	164 (0.9)	114 (0.7)	50 (2.7)			158 (0.9)	6 (2.7)
Multiple drugs	19 (0.1)	16 (0.1)	3 (0.2)			19 (0.1)	0
**Specialty of the prescribing physician**							
Child and adolescent psychiatrist	9,460 (50.6)	8,751 (51.9)	709 (38.8)	9,267 (50.9)	193 (39.8)	9,388 (50.8)	72 (32.7)
Neurologist/psychiatrist	111 (0.6)	98 (0.6)	13 (0.7)	103 (0.6)	8 (1.6)	111 (0.6)	0
Pediatrician	4,399 (23.5)	4,080 (24.2)	319 (17.4)	4,266 (23.4)	133 (27.4)	4,330 (23.4)	69 (31.4)
General practitioner	407 (2.2)	353 (2.1)	54 (3.0)	394 (2.2)	13 (2.7)	400 (2.2)	7 (3.2)
Other specialty	263 (1.4)	232 (1.4)	31 (1.7)	260 (1.4)	3 (0.6)	259 (1.4)	4 (1.8)
Unknown	4,063 (21.7)	3,360 (19.9)	703 (38.4)	3,928 (21.6)	135 (27.8)	3,995 (21.6)	68 (30.9)
**Psychiatric hospitalization**	2,168 (11.6)	2,001 (11.9)	167 (9.1)	2,035 (11.2)	133 (27.4)	2,126 (11.5)	42 (19.1)
**Psychiatric comorbidities**							
Conduct disorders (F90.1, F91, and F92)	6,722 (35.9)	6,369 (37.7)	353 (19.3)	6,488 (35.6)	234 (48.2)	6,612 (35.8)	110 (50.0)
Emotional disorders in childhood and anxiety	4,500 (24.1)	4,161 (24.7)	339 (18.5)	4,374 (24.0)	126 (26.0)	4,468 (24.2)	32 (14.5)
(F40, F41.0, F41.1, F41.3, F41.8, F41.9, and							
F93)							
Disorders of social functioning (F94)	849 (4.5)	777 (4.6)	72 (3.9)	819 (4.5)	30 (6.2)	837 (4.5)	12 (5.5)
Reactions to severe stress (F43.0, F43.1,	724 (3.9)	646 (3.8)	78 (4.3)	697 (3.8)	27 (5.6)	716 (3.9)	8 (3.6)
F43.8, and F43.9)							
Mental retardation (F70–F79)	593 (3.2)	508 (3.0)	85 (4.6)	549 (3.0)	44 (9.1)	577 (3.1)	16 (7.3)
Depression (F32, F33, F41.2, and F43.2)	3,151 (16.8)	2,820 (16.7)	331 (18.1)	3,055 (16.8)	96 (19.8)	3,136 (17.0)	15 (6.8)
Tic disorders (F95)	679 (3.6)	628 (3.7)	51 (2.8)	637 (3.5)	42 (8.7)	672 (3.6)	7 (3.2)
Substance use disorders (F10–F19)	163 (0.9)	132 (0.8)	31 (1.7)	155 (0.9)	8 (1.6)	161 (0.9)	2 (0.9)
Somatoform disorders (F45)	1,136 (6.1)	1,029 (6.1)	107 (5.9)	1,092 (6.0)	44 (9.1)	1,130 (6.1)	6 (2.7)
Sleep disorders (F51, G47)	848 (4.5)	726 (4.3)	122 (6.7)	810 (4.4)	38 (7.8)	812 (4.4)	36 (16.4)
Specific developmental disorders of speech	4,365 (23.3)	3,941 (23.4)	424 (23.2)	4,252 (23.3)	113 (23.3)	4,239 (22.9)	126 (57.3)
and language (F80)							
Specific developmental disorders of	4,331 (23.2)	4,074 (24.1)	257 (14.1)	4,246 (23.3)	85 (17.5)	4,330 (23.4)	1 (0.5)
scholastic skills (F81)							
Specific developmental disorder of motor	3,109 (16.6)	2,876 (17.0)	233 (12.7)	3,044 (16.7)	65 (13.4)	3,039 (16.4)	70 (31.8)
function (F82)							
Mixed specific developmental disorders (F83)	2,150 (11.5)	1,933 (11.5)	217 (11.9)	2,075 (11.4)	75 (15.5)	2,078 (11.2)	72 (32.7)
Pervasive developmental disorders (F84.0,	1,107 (5.9)	918 (5.4)	189 (10.3)	1,012 (5.6)	95 (19.6)	1,069 (5.8)	38 (17.3)
F84.1, F84.5, F84.8, and F84.9)							
Non-organic enuresis and/or encopresis	1,329 (7.1)	1,230 (7.3)	99 (5.4)	1,289 (7.1)	40 (8.2)	1,319 (7.1)	10 (4.5)
(F98.0, F98.1)							
**Number of psychiatric comorbidities^#^**							
0	3,347 (17.9)	2,927 (17.3)	420 (23.0)	3,293 (18.1)	54 (11.1)	3,325 (18.0)	22 (10.0)
1	5,120 (27.4)	4,566 (27.1)	554 (30.3)	5,007 (27.5)	113 (23.3)	5,081 (27.5)	39 (17.7)
2	4,591 (24.5)	4,178 (24.8)	413 (22.6)	4,478 (24.6)	113 (23.3)	4,532 (24.5)	59 (26.8)
3+	5,645 (30.2)	5,203 (30.8)	442 (24.2)	5,440 (29.9)	205 (42.3)	5,545 (30.0)	100 (45.5)
**History of other psychopharmacological drugs**							
Antipsychotics (N05A)	532 (2.8)	415 (2.5)	117 (6.4)	463 (2.5)	69 (14.2)	510 (2.8)	22 (10.0)
Anxiolytics (N05B)	90 (0.5)	66 (0.4)	24 (1.3)	83 (0.5)	7 (1.4)	82 (0.4)	8 (3.6)
Hypnotics and sedatives (N05C)	198 (1.1)	158 (0.9)	40 (2.2)	176 (1.0)	22 (4.5)	178 (1.0)	20 (9.1)
Antidepressants (N06A)	326 (1.7)	237 (1.4)	89 (4.9)	303 (1.7)	23 (4.7)	325 (1.8)	1 (0.5)

*Values are numbers (percentages)*.

*ADHD, attention deficit hyperactivity disorder; ATX, atomoxetine; DEX, dexamfetamine; GUA, guanfacine; LDX, lisdexamfetamine; MPH, methylphenidate*.

+ *Second-line drugs: LIS, DEX, GUA, or multiple drugs (including MPH and/or ATX if not used as monotherapy); first-line: MPH or ATX*.

# *Exclusively related to the 16 above-mentioned comorbidities*.

More than half of all users received the first prescription from a child and adolescent psychiatrist and almost one quarter received the prescription from a pediatrician. The most frequent psychiatric comorbidities were conduct disorders and emotional disorders in childhood or anxiety; about 80% had at least one comorbidity. With regard to history of other psychopharmacological drugs, antipsychotics were most frequently prescribed.

### Lack of a Documented ADHD Diagnosis at First-Time Use of ADHD Medication: Patient and Prescriber Characteristics

Psychiatric hospitalizations occurred less often in first-time users without than in those with a documented ADHD diagnosis (9 vs. 12%; Table 1). Most psychiatric comorbidities were less prevalent in individuals with a lack of a documented ADHD diagnosis. For example, conduct disorders were recorded in 19% of first-time users without and in 38% of those with a documented ADHD diagnosis.

### Second-Line Drug as First ADHD Medication: Patient and Prescriber Characteristics

In recipients of second-line ADHD medication, there was more often a lack of ADHD diagnosis compared with individuals receiving first-line ADHD medication (Table 1). The percentage of prescribing pediatricians was higher among first-time users of second-line ADHD medication as compared to first-time users of first-line drugs.

The results from the multivariable logistic regression model are shown in Table 2. The following characteristics were associated with an off-label prescription of a second-line ADHD medication: Compared with adolescents aged 12–17 years, patients aged below 6 years were more likely to receive a second-line drug. Further, individuals were more likely to receive second-line ADHD medication if they received the prescription from a pediatrician; had a psychiatric hospitalization; were diagnosed with conduct disorders, mental retardation, tic disorders or pervasive developmental disorders; or had a history of antipsychotics.

**Table 2:** Adjusted odds ratios for characteristics associated with receiving a second-line drug among first-time users of ADHD medication.

**Characteristic**	Adjusted odds ratios (95% CI) for receiving second-line as compared to receiving first-line drug^*^
**Male sex (Ref.: female)**	0.84 (0.68–1.04)
**Age group in years**	
<6	**1.70 (1.02–2.85)**
6–11	**0.78 (0.71–0.86)**
12–17	Ref.
**Specialty of the prescribing physician**	
Child and adolescent psychiatrist	Ref.
Pediatrician	**1.56 (1.23–1.97)**
Other specialty/unknown	1.16 (0.92–1.47)
**Lack of a documented ADHD diagnosis (Ref.: No)**	**2.10 (1.63–2.69)**
**Psychiatric hospitalization (Ref.: No)**	**1.90 (1.48–2.43)**
**Psychiatric comorbidities (Ref.: No)**	
Conduct disorders	**1.54 (1.26–1.88)**
Emotional disorders in childhood and anxiety	0.99 (0.80–1.24)
Mental retardation	**1.54 (1.07–2.22)**
Depression	0.91 (0.71–1.17)
Tic disorders	**2.08 (1.47–2.94)**
Somatoform disorders	1.23 (0.89–1.72)
Pervasive developmental disorders	**2.88 (2.24–3.72)**
**History of other psychopharmacological drugs (Ref.: No)**	
Antipsychotics	**2.80 (2.05–3.84)**
Antidepressants	1.29 (0.78–2.12)

*ADHD, attention deficit hyperactivity disorder; CI, confidence interval*.

*Boldface indicates statistical significance*.

* *Second-line drug: lisdexamfetamine, dexamfetamine, guanfacine, or multiple drugs (including methylphenidate and/or atomoxetine if not used as monotherapy); first-line: methylphenidate or atomoxetine. The logistic regression model is adjusted for all variables in this table*.

### ADHD Medication Prescriptions in Children <6 Years: Patient and Prescriber Characteristics

In patients aged below 6 years, second-line ADHD medication use was more prevalent (Table 1). They also more often had no documented ADHD diagnosis and a pediatrician as the prescribing physician. Among others, conduct disorders and pervasive developmental disorders were more frequent in this patient group. Among all first-time users younger than 6 years (*n* = 220), 10% (*n* = 22) were aged 3 years or younger.

## Discussion

This study evaluated adherence to prescribing guidelines in first-time users of ADHD medication among children and adolescents in routine care in Germany based on three key off-label criteria. Our main finding is that prescribing ADHD medication without a confirmed ADHD diagnosis was relatively common, while rather few first users received prescriptions of a second-line ADHD medication, and only a small percentage of users was aged below 6 years.

### Lack of a Documented ADHD Diagnosis

During the study period, ADHD and narcolepsy were the only licensed indications for each of the ADHD medications assessed in this study (14). As expected, narcolepsy without ADHD was documented in very few cases only. We therefore focused on the off-label criterion indicating a lack of a documented diagnosis of ADHD.

A prior study on LDX used data on prescriptions and diagnoses of ADHD from eight European countries (15). The main results include that about 62–95% of pediatric and adult LDX first-time users had a recorded diagnosis of ADHD (15). Although the comparability is limited due to the focus on LDX users only, this result is in accordance with our finding.

Given that current clinical guidelines consistently recommend a structured, comprehensive ADHD assessment (6, 9) and that German regulations regarding the reimbursement of stimulants require detailed diagnostics (10), it is striking that in our study about one in 10 patients had no documented diagnosis of ADHD at the time of the first prescription of an ADHD drug. As we reviewed recorded diagnoses from up to 2 years before the first prescription and from any provider—including psychotherapists—we do not believe that recorded diagnoses of ADHD before the first drug treatment or during a prior non-pharmacological treatment have been overlooked.

The characteristics of patients with a lack of a documented ADHD diagnosis do not indicate that they were more severe cases warranting immediate drug treatment. Characteristics such as psychiatric hospitalizations as well as comorbidities that would indicate a more complex psychopathology (e.g., conduct disorders) were even less frequent in first-time users of ADHD drugs with a lack of a documented ADHD diagnosis as compared to those with a diagnosis of ADHD.

We conclude that prescribing ADHD medication to children and adolescents without a clinically confirmed ADHD diagnosis was relatively common. Assuming that in these cases diagnostic requirements were not met, our results indicate irrational prescribing with possible associated consequences such as exposing the patient to unnecessary risks of side effects.

### Second-Line Drug as First ADHD Medication

In our study, characteristics of patients who used second-line as compared to those who used first-line drugs as the first ADHD medication differed markedly. These characteristics—higher prevalence of prior psychiatric hospitalization and of antipsychotic prescription, conduct disorders, mental retardation, tic disorders, and pervasive developmental disorders—indicate a more complex and extensive psychopathology in these patients. In the multivariable regression, these characteristics were positively associated with receiving a second-line drug.

In addition to the above-mentioned characteristics indicating a more complex clinical presentation, both children below 6 years as well as those receiving the prescription from a pediatrician were also more likely to be prescribed a second-line drug as their first ADHD medication.

Regarding potential reasons for starting ADHD treatment with second-line medication, prescribing based on trial data ahead of formal licensing might be a potential cause. Nevertheless, clinical data on efficacy do not support the superiority of LDX, DEX, or GUA (i.e., second-line drugs in this study) over MPH or ATX (i.e., first-line drugs in this study) for patients with the above-mentioned conditions (16). Considering data from randomized controlled trials on tolerability, amphetamines (including LDX and DEX) and GUA—but not MPH—were inferior to placebo in children and adolescents (17). According to network meta-analyses from head-to-head trials in pediatric patients diagnosed with ADHD, LDX was more likely to cause some serious side effects, including sleep disorders and irritability (18). Finally, large post-authorization safety studies evaluating rare outcomes and prescribing in routine care are scarce for LDX, DEX, and GUA—particularly as compared with the abundance of such studies for MPH.

The fact that children aged below 6 years were more likely to receive a second-line drug as the first ADHD medication was especially surprising. A possible reason might be that prescribers estimate the risk of adverse events of second-line ADHD drugs as less than MPH. Yet, such an attitude is not supported by current evidence: To date, there are only few studies assessing safety and efficacy of ADHD medication in children younger than 6 years. This is particularly true for the second-line drugs assessed in this study (i.e., LDX, DEX, and GUA). It is expected that the evidence base on ADHD medication for children younger than 6 years will improve soon as numerous randomized controlled trials are planned or currently running (19). However, second-line drugs should not be preferably prescribed—particularly to preschool children—as long as superiority over first-line drugs is not proven by sound evidence. Currently, MPH is considered the treatment of first choice for preschool children, if pharmacotherapy is indicated, as it is the ADHD drug with the strongest evidence for efficacy and safety in this population (16, 20).

Surprisingly—even when adjusted for age and characteristics indicating the complexity of ADHD cases—patients were more likely to receive a second-line drug as the first ADHD medication when the prescription was made by a pediatrician. This is particularly remarkable as more severe ADHD cases are usually pharmacologically treated by specialized child and adolescent psychiatrists, as was suggested by a previous study (21). Further research is needed to evaluate this potentially irrational off-label prescribing.

### ADHD Medication in Patients Below 6 Years

One study based on UK data found that in a sample of individuals aged below 16 years, 4% of ADHD medication users were aged below 6 years. This percentage is somewhat higher than in our study. Notably, the study was limited to the years 1992–2013, and the more recently approved drugs LDX and GUA were not included (22). Two other European studies, which also presented findings on guideline/label adherence, only evaluated one specific drug—MPH (23) or LDX (15). The study on MPH, based on French prescription data, found that 5% of incident MPH users below 18 years were aged younger than 6 years (23). This is also higher than in our study and might indicate that off-label prescribing to children below 6 years is less common in Germany than in other European countries. The second study on LDX found that fewer than 1% of pediatric and adult LDX users were younger than 6 years (15). This is in accordance with our findings. As discussed earlier, the percentages for receiving second-line ADHD medication were higher in patients aged below 6 years as compared to those of higher age although there is a lack of evidence supporting the superiority of these drugs in preschool children (19). It is surprising that more than twice as often no ADHD diagnosis was documented in children below 6 years than in older children. Given the fact that studies on ADHD medication for children aged below 6 years are scarce (19), a comprehensive assessment of the ADHD diagnosis—as consistently recommended by clinical guidelines (6, 9)—should be self-evident as a crucial element of the risk-benefit assessment.

One explanation for not finding recorded diagnoses might be concerns of physicians and/or parents regarding a potential stigmatization of children with ADHD (24). This could have led to a reluctance to diagnose the disorder, particularly in preschool children with regard to school entry. However, as far as public beliefs are concerned, treatment with ADHD medication is even less commonly accepted than the diagnosis (25).

In patients aged below 6 years as compared with older patients, prescribers were less often specialists and more often pediatricians. However, these results do not allow conclusions about the prescribers' specialty or their preferences regarding prescribing off-label to children younger than 6 years as the proportion of individuals with contact to specialists for mental health disorders might be much smaller for younger patients. This was shown for pediatric patients diagnosed with autism spectrum disorders in Germany (26). However, the German guideline on ADHD recommends that drug treatment to preschool children should only be prescribed by a physician with special knowledge of behavioral disorders in this age group (11). Unfortunately, we do not know whether the pediatricians who prescribed drugs to preschool children in our study have this knowledge—in contrast to child and adolescent psychiatrists, who are—by training—best qualified to do so.

The German guideline on ADHD (11)—similar to other guidelines from the UK (27) and US (20)—recommends parent training and/or interventions in kindergarten/school as the first line of treatment in children younger than 6 years. As a caveat in our study, it is unknown whether any of these interventions had been used prior to initiating drug treatment or whether they had proven ineffective, which would justify initiating ADHD medication.

Similar to recipients of second-line drugs, first-time users aged below 6 years more often had psychiatric hospitalizations, conduct disorders, pervasive developmental disorders, and history of antipsychotics, i.e., characteristics indicating more complex clinical presentations. Guidelines do not preclude off-label prescribing to children aged below 6 years in severe cases and after individual risk-benefit assessment. In fact, the guidelines from the UK National Institute for Health and Care Excellence (NICE) differentiate between the age groups below 5 years and older (27). Against this background, it is encouraging that most patients younger than 6 years in our study were aged between 4 and 5 years, i.e., age groups that are not precluded from receiving ADHD medication. However, we found physicians who prescribed ADHD medication to children aged 3 years or younger in our study, which indicates irrational prescribing as there is a lack of evidence in this age group—regarding both, diagnosis and drug treatment.

### Implications

In routine care in Germany, adherence to prescribing guidelines is suboptimal in a substantial proportion of children and adolescents initiating medication to treat ADHD. Physicians should follow current guideline recommendations on ADHD to optimize rational prescribing and avoid adverse events such as insomnia, seizures, tics, loss of appetite, and possible growth deficits. This holds especially true for some pediatricians, who appear to be susceptible to non-adherence to guidelines or label requirements—at least with regard to prescribing second-line drugs as first ADHD medication.

Due to potential side effects and frequent use of drugs to treat ADHD, future research should continue to monitor their off-label use in children and adolescents. Evaluating prescribing behavior following the release of the new German ADHD guidelines in 2018 will provide information for further measures aimed at implementing evidence-based recommendations in routine care.

### Strengths and Limitations

The main strength of this study is that the underlying routinely collected prescription data are not prone to both, non-responder and recall bias. This is particularly important as it cannot be assumed that prescribers would admit if they did not adhere to prescribing guidelines. In addition, we used a large statutory health insurance database covering about one fifth of the German population. Among children and adolescents in Germany, prevalence of drug use does not differ substantially between different types of statutory health insurance providers (28). We therefore believe that the results of this study are representative for patients covered by statutory health insurance in Germany, who account for almost 90% of the general population (13). In our study, we explicitly focused on children and adolescents who were first-time users of any of the ADHD medications available, which allowed us to assess off-label first-time use of second-line drugs. In contrast to other studies, we considered important clinical information, such as psychiatric hospitalizations, comorbidities, and other psychopharmacological drugs.

A general limitation of claims data is that the validity of outpatient diagnoses is suboptimal; also, ADHD in children might be overdiagnosed in Germany (29). This, however, is not a relevant limitation in our study as overdiagnosis would rather lead to underestimating the proportion of first-time users with a lack of an ADHD diagnosis. Although we used information on psychiatric hospitalizations and comorbidities as a proxy for the complexity of ADHD cases, this study is limited by a lack of information on the severity of ADHD. A further limitation, particularly regarding the outcome of ADHD medication in patients below 6 years, is the lack of information on prior non-pharmacological interventions.

## Conclusions

Our findings show that in more than 10% of pediatric first-time ADHD medication users, prescribers did not adhere to prescribing guidelines. Initiating ADHD drugs without a confirmed ADHD diagnosis was the most common of the three studied off-label criteria. We found off-label use in terms of drug choice and age of patients in a small percentage of pediatric first-time users of drugs to treat ADHD. Since ADHD medication is prescribed frequently in children and adolescents, improving rational prescribing in this area is of high relevance for public health.

## Data Availability Statement

As we are not the owners of the data, we are not legally entitled to grant access to the data of the German Pharmacoepidemiological Research Database. In accordance with German data protection regulations, access to the data is granted only to BIPS employees on the BIPS premises and in the context of approved research projects. Third parties may only access the data in co-operation with BIPS and after signing an agreement for guest researchers at BIPS. Requests to access the datasets should be directed to OS, scholle@leibniz-bips.de.

## Ethics Statement

In Germany, the utilization of health insurance data for scientific research is regulated by the Code of Social Law. All involved health insurance providers as well as the German Federal Office for Social Security and the Senator for Health, and Women and Consumer Protection in Bremen as their responsible authorities approved the use of GePaRD data for this study. Informed consent for studies based on claims data is required by law unless obtaining consent appears unacceptable and would bias results, which was the case in this study. According to the Ethics Committee of the University of Bremen, studies based on GePaRD are exempt from institutional review board review.

## Author Contributions

OS and CB conceptualized the research question. OS wrote the first draft of the manuscript. BK, OR, and CB revised it critically for important intellectual content and approved the final version to be published. All authors designed the study and participated in the discussion and interpretation of the results.

## Conflict of Interest

OS, BK, and OR are working at an independent, non-profit research institute, the Leibniz Institute for Prevention Research and Epidemiology – BIPS. Unrelated to this study, BIPS occasionally conducts studies financed by the pharmaceutical industry. Almost exclusively, these are post-authorization safety studies (PASS) requested by health authorities. The design and conduct of these studies as well as the interpretation and publication are not influenced by the pharmaceutical industry. The study presented was not funded by the pharmaceutical industry. The remaining author declares that the research was conducted in the absence of any commercial or financial relationships that could be construed as a potential conflict of interest.
